# Antiviral Activity of Selective Estrogen Receptor Modulators against Severe Fever with Thrombocytopenia Syndrome Virus In Vitro and In Vivo

**DOI:** 10.3390/v16081332

**Published:** 2024-08-20

**Authors:** Xintong Yan, Chongda Luo, Jingjing Yang, Zhuang Wang, Yunfeng Shao, Ping Wang, Shaokang Yang, Yuexiang Li, Qingsong Dai, Wei Li, Xiaotong Yang, Huimin Tao, Sichen Ren, Zhenyang Li, Xiaojia Guo, Siqi Li, Weiyan Zhu, Yan Luo, Jiazheng Li, Song Li, Ruiyuan Cao, Wu Zhong

**Affiliations:** 1 School of Pharmaceutical Sciences, Hainan University, Haikou 570228, China; yanxintong@hainanu.edu.cn (X.Y.); yangjingjing@hainanu.edu.cn (J.Y.); wp1303544657@163.com (P.W.); rnschn@yeah.net (S.R.); ljz18743426877@163.com (J.L.);; 2 Song Li’s Academician Workstation, School of Pharmaceutical Sciences, Hainan University, Yazhou Bay, Sanya 572000, China; 3 National Engineering Research Center for the Emergency Drug, Beijing Institute of Pharmacology and Toxicology, Beijing 100850, China; todd1997@mail.nwpu.edu.cn (Z.W.); ysk120650@163.com (S.Y.); lyx1986528@126.com (Y.L.); qingsong4321@126.com (Q.D.); a_moon1096@163.com (W.L.); yangxiao8792@163.com (X.Y.); thm21@mails.tsinghua.edu.cn (H.T.); lzy910722@126.com (Z.L.); 15227117791@163.com (X.G.); juneaew@163.com (S.L.); zwyee123456@163.com (W.Z.)

**Keywords:** severe fever with thrombocytopenia syndrome virus, selective estrogen receptor modulator, bazedoxifene acetate, antiviral

## Abstract

Severe fever with thrombocytopenia syndrome virus (SFTSV), also known as the Dabie Banda virus, is an emerging tick-borne Bunyavirus that causes severe fever with thrombocytopenia syndrome (SFTS). Currently, symptomatic treatment and antiviral therapy with ribavirin and favipiravir are used in clinical management. However, their therapeutical efficacy is hardly satisfactory in patients with high viral load. In this study, we explored the antiviral effects of selective estrogen receptor modulators (SERMs) on SFTSV infection and the antiviral mechanisms of a representative SERM, bazedoxifene acetate (BZA). Our data show that SERMs potently inhibited SFTSV-induced cytopathic effect (CPE), the proliferation of infectious viral particles, and viral RNA replication and that BZA effectively protected mice from lethal viral challenge. The mode of action analysis reveals that BZA exerts antiviral effects during the post-entry stage of SFTSV infection. The transcriptome analysis reveals that GRASLND and CYP1A1 were upregulated, while TMEM45B and TXNIP were downregulated. Our findings suggest that SERMs have the potential to be used in the treatment of SFTSV infection.

## 1. Introduction

Severe fever with thrombocytopenia syndrome (SFTS) is a life-threatening infection caused by a novel bunyavirus, SFTS virus (SFTSV). This enveloped RNA virus was later reclassified as the Dabie Banda virus. According to the International Committee on the Taxonomy of Viruses, the virus belongs to the *Bandavirus* genus, *Phenuiviridae* family, *Hareavirales* order, and *Bunyaviricetes* class [1]. It was first identified in China in 2009 [2]. To date, Asian countries, including China [3], South Korea [4], Japan [5], Vietnam [6], and Myanmar [3], have reported confirmed cases, with an overall case fatality rate (CFR) ranging from 12% to 50%. China has reported the majority of SFTS cases, with 13,824 cases from 25 provinces as of the end of 2019, of which 8899 were laboratory-confirmed. Moreover, similar cases caused by the heartland virus, another emerging pathogen that is genetically closely related to SFTSV, were found in North America, suggesting that SFTS and SFTS-like diseases may disseminate worldwide [7].

To date, no specific antiviral drugs have been approved for treating SFTSV infections. Ribavirin inhibits SFTSV in vitro. The combination of type-I/II interferons (IFNs) with ribavirin has reduced SFTSV infection and may, therefore, contribute to the clinical treatment of patients with SFTS [8,9,10]. Nevertheless, no significant change in the CFR was observed in patients who received ribavirin compared with the control group, regardless of the initial viral load [11]. Favipiravir (T-705) has broad-spectrum anti-RNA virus activity, and both in vivo and in vitro anti-SFTSV activities have been reported [12]. A single-blinded randomized controlled clinical trial was conducted to assess the efficacy and safety of T-705 in treating SFTS [13]. The T-705-treated group showed a shorter interval of viral clearance, lower incidence of hemorrhagic signs, and faster recovery of laboratory abnormalities compared with the controls. Nifedipine, a calcium channel blocker (CCB), inhibited SFTSV replication in vitro and in vivo [14]. Importantly, by performing a retrospective clinical investigation on a large cohort of 2087 patients with SFTS, researchers found that nifedipine administration enhanced viral clearance, improved clinical recovery, and reduced the CFR. However, this study was subject to the limitations that the clinical results of patients with SFTS were retrospective, with no randomization, and potentially flawed. Further clinical investigations of the potential therapeutic effect of CCBs against SFTS should also thoroughly evaluate factors, including the potential drug side effects, drug–drug interactions, and in vivo drug concentration. To date, no licensed vaccines or effective therapeutics have been developed against SFTSV.

Selective estrogen receptor modulators (SERMs) are anti-estrogens that are designed to compete with estrogen and modulate estrogen receptor (ER) activity by altering the cofactors with which it associates [15,16,17]. Based on their chemical structure, SERMs can be classified into triphenylethylenes (tamoxifen, clomiphene, and toremifene), benzothiophenes (raloxifene and arzoxifene), phenylindoles (bazedoxifene and pipendoxifene), and tetrahydronaphthalenes (lasofoxifene) (Figure S1). Studies have shown that SERMs exert antiviral effects. During the COVID-19 pandemic, clomifene citrate, tamoxifen, toremifene citrate, and bazedoxifene were reported to possess excellent antiviral activity against coronavirus in several cellular infection models and a hamster infection model. Also, they differentially reduced the expression of IL-6 mRNA in hamster lungs. In particular, bazedoxifene acetate (BZA) was identified to act on the penetration stage of the post-attachment step via altering cholesterol distribution and endosome acidification [18]. The Ebola virus (EBOV) is another lethal pathogen that causes hemorrhagic fever syndrome. A study showed that clomiphene and toremifene act as potent inhibitors of EBOV infection in vitro and in vivo by preventing the early stages of viral infection, particularly the viral–host membrane fusion process. This anti-EBOV activity occurred even in the absence of detectable estrogen receptor expression, suggesting that clomiphene and toremifene are not working through the classical pathways associated with the estrogen receptor. The response appeared to be an off-target effect [19,20].

In this study, we explored the anti-SFTSV effects of SERMs in vitro and in vivo and the antiviral mechanisms of a favorable compound, BZA. Robust antiviral activities were revealed, and mode of action studies suggest that BZA inhibited the post-entry stage of SFTSV infection. Transcriptome analysis suggests that Glycosaminoglycan Regulatory Associated Long Non-coDing RNA (GRASLND) and Cytochrome P450 1A1 enzyme (CYP1A1) were upregulated, while Transmembrane protein 45B (TMEM45B) and Thioredoxin-interacting protein (TXNIP) were downregulated. Our findings suggest the potential use of SERMs as countermeasures against SFTSV infection.

## 2. Materials and Methods

### 2.1. Cell Culture and Virus Propagation

Huh7 and Huh7.5 cells were cultured in Dulbecco’s Modified Eagle’s Medium (DMEM) (GIBCO BRL, Grand Island, NY, USA) supplemented with 10% fetal bovine serum (FBS) (GIBCO BRL, Grand Island, NY, USA) and 1% penicillin–streptomycin (Thermo Fisher Scientific, Waltham, MA, USA). All cells were cultured at 37 °C with 5% CO_2_ in a humidified incubator. SFTSV was isolated from clinical samples in our laboratory (GenBank: MZ561690.1, MZ561691.1, and MZ561692.1) [21]. SFTSV was cultured in the Huh7 cell until cytopathy was observed. The medium was subsequently centrifuged at 4000 rpm for 10 min to remove the cellular debris and obtain the viral supernatant. The viral titers were determined using a 50% tissue culture infectious dose (TCID_50_) assay on the Huh7 and Huh7.5 cells, and the virus stock was stored at −80 °C. We are grateful to Dr. Zhihai Chen (Beijing Ditan Hospital, Capital Medical University, Beijing, China) for kindly providing us with the clinical samples collected from SFTSV patients. All cellular experiments were performed in an enhanced Biosafety Level 2 (BSL-2+) laboratory. All animal experiments were performed in an Animal Biosafety Level 3 (ABSL-3) laboratory.

### 2.2. Compounds

Acolbifene HCl, Arzoxifene HCl, and T-705 were purchased from MedChemExpress (Shanghai, China). Lasoxifene tartrate, Amcenestrant, Brilanestrant, Clomiphene citrate, Fulvestrant, Cyclofenil, Chlorotrianisene, Tamoxifen citrate, Estrio, and Pipendoxifene HCl were purchased from TargetMol Chemicals (Shanghai, China). Raloxifene HCl, G-1, Camizestrant, Endoxifene HCl, and BZA were purchased from Selleck Chemicals (Shanghai, China). The catalog numbers of the compounds are listed in Table S1. All compounds were dissolved in dimethylsulfoxide (DMSO) as 100 mM stock and stored at −20 °C.

### 2.3. Cytopathic Effect Inhibition Assay

In previous work, our laboratory constructed a screening model for anti-SFTSV compounds [21]. Cells were seeded in 96-well white plates and incubated at 37 °C and 5% CO_2_. The medium was removed and replenished with three-fold serially diluted compounds and 100 × TCID_50_ SFTSV the following day. A virus-free medium was added to the mock-infected wells. T-705 was used as a positive control for CPE inhibition in SFTSV. Subsequently, these plates were incubated until CPE was achieved within 5–7 days, depending on the virus. The cell viability of each group was assessed using a CellTiter-Glo cell viability assay and a SpectraMax M5 microplate reader (Molecular Devices, San Jose, CA, USA). The data were fitted using Origin 9.0 Software to calculate the half-maximal effect concentration (EC_50_) and concentration of cytotoxicity 50% (CC_50_) values for inhibitory efficiency and cytotoxicity. These values were subsequently used to derive the selectivity index (SI) value for each compound (SI = CC_50_/EC_50_).

### 2.4. Quantitative Real-Time PCR (qRT-PCR)

Huh7 cells were seeded in 12-well plates at a density of 1.0 × 10^5^ cells/well and cultured overnight. The cells were then infected with SFTSV at a multiplicity of infection (MOI) of 0.1 with indicated concentrations of tested compounds. After 48 h of incubation, total RNA was extracted from the cell samples using TRIzol reagent (Invitrogen, Waltham, MA, USA). qRT-PCR was performed using One Step PrimeScript™ RT-PCR Kit (TaKaRa, Otsu, Shiga, Japan), according to the manufacturer’s instructions using primers and probes specific for SFTSV (Forward primer: GGGTCCCTGAAGGAGTTGTAAA; reverse primer: TGCCTTCACCAAGACTATCAATGT; and probe: TTCTGTCTTGCTGGCTCCGCGC). Viral RNA copy numbers were calculated using the cycle threshold value obtained for each sample and compared to a known copy number standard curve. The SFTSV standard plasmid with pUC57 as the vector was synthesized by Sangon Biotech (Shanghai, China) according to the above primer probe sequences and solubilized in RNase-free water. The copy number of the standard was calculated according to the concentration and molecular weight of the plasmid. The supernatant was collected for the quantification of infectious viral particles using a plaque formation assay.

### 2.5. Plaque Assay (Plaque-Forming Unit [PFU] Assay)

Huh7 cells seeded in 12-well plates were incubated with the serial 10-fold dilutions of the samples for 2 h and then washed twice with PBS. Plaque fluid consisting of 2% low-melting-point agarose and 2 × DMEM (1:1 vol/vol) was added to the plates, and the cells were cultured for four days. PFUs were counted after staining the cells with crystal violet solution at room temperature for 10 min. Data were obtained from three independent experiments.

### 2.6. Mouse Experiments

In previous work, our laboratory constructed several mouse models for evaluating anti-SFTSV compounds [21]. Balb/c mice viremia: The 6-week-old Balb/c female mice (Beijing Vital River Laboratory Animal Technology, Beijing, China) were randomly distributed into five groups. Each group comprised eight mice. Four groups of mice were subjected to an intraperitoneal injection with 1 × 10^5^ PFU/mouse of SFTSV. One group that did not undergo viral infection served as a normal control. Mice in groups 1–4 received different compounds at appropriate doses: vehicle, BZA (30 mg/kg), BZA (10 mg/kg), or BZA (3 mg/kg). After setting the time of infection to 0 h, BZA was administered by oral gavage at −12 h, 0 h, and 12 h. At 24 h post-infection, blood from the orbital venous plexus was collected from all mice and subsequently tested for viral load.

A129 mice viremia: The *Ifnar^−/−^* A129 mice weighing about 20 g (6–8 weeks old; a gift from the State Key Laboratory of Pathogen and Biosecurity, Beijing Institute of Microbiology and Epidemiology) were randomly distributed into four groups. Each group comprised eight mice. Three groups of mice were injected intraperitoneally with 50 PFU/mouse of SFTSV. Mice in groups 1–4 received different compounds at appropriate doses: vehicle, BZA (30 mg/kg), BZA (10 mg/kg), or BZA (3 mg/kg). Setting the time of infection at 0 h, BZA was administered by oral gavage at −12 h, 0 h, and 12 h. At 24 h post-infection, blood from the orbital venous plexus was collected from all mice and subsequently tested for viral load.

For the mouse lethal protection model, 25 μL of DMEM containing 1.25 × 10^6^ PFU of SFTSV was injected intraperitoneally into one-day-old ICR suckling mice. Either BZA or the vehicle was administered intraperitoneally after 4 h, and the treatments (20, 10, and 5 mg/kg) were administered daily for seven days. The survival and body weight of the mice were then monitored for 21 days, while the other experimental groups were the same as previously mentioned, but the administration mode was changed to oral gavage in the mother mice.

### 2.7. Ethics Statement

All mice were maintained under specific pathogen-free conditions, and all animal experiments were approved by the Institutional Animal Care and Use Committee of the Beijing Institute of Pharmacology and Toxicology.

### 2.8. Time-of-Addition Assays

Huh7 cells were either pre-treated, co-treated, or post-treated with BZA (3 μM) during SFTSV infection. In brief, (I) Pre-treatment: Huh7 cells were inoculated with BZA at 37 °C for 2 h and subsequently washed thrice with PBS to remove BZA, then the cells were infected with SFTSV (MOI = 0.1) for 2 h and subsequently washed thrice with PBS to remove unbound viruses. (II) Co-treatment: Huh7 cells were simultaneously treated with SFTSV (MOI = 0.1) and BZA at 37 °C for 2 h, then the cells were washed thrice with PBS to remove BZA and unbound viruses. (III) Post-treatment: Huh7 cells were infected with SFTSV (MOI = 0.1) at 37 °C for 2 h and subsequently washed thrice with PBS, then the cells were treated with BZA at 37 °C. (IV) Whole stage treatment: Huh7 cells were simultaneously treated with SFTSV (MOI = 0.1) and BZA at 37 °C for 2 h, then the cells were washed thrice with PBS to remove unbound viruses. As the compound was also removed, the same working concentration of BZA was added. All these treated cells were then further cultured at 37 °C for 24 h. T-705, a compound that has been reported to act in the post-entry phase, was used as a positive control in this experiment.

### 2.9. Transcriptome Analysis

Huh7 cells were incubated with BZA or DMSO (0.1%) and SFTSV for 24 h at 37 °C in DMEM with 2% FBS and 1% penicillin–streptomycin. The MOI was 0.1. Total RNA was extracted using TRIzol reagent (Invitrogen). RNA purity was assessed using the Nanodrop One. RNA purification, reverse transcription, library construction, and sequencing were performed at Shanghai Majorbio Bio-pharm Biotechnology Co., Ltd. (Shanghai, China), according to the manufacturer’s instructions (Illumina, San Diego, CA, USA). Data were analyzed using the online Majorbio Cloud Platform (www.majorbio.com, accessed on 20 December 2023).

### 2.10. Statistical Analysis

All data were presented as the mean ± SD from at least three independent experiments and analyzed using GraphPad Prism 7 software. An unpaired two-tailed Student’s *t*-test or one-way analysis of variance was used to analyze the statistical significance of two or multiple groups, respectively. The survival curve was determined using the log-rank test. The two configurations were deemed to be statistically significant if *p* < 0.05 (*), *p* < 0.01 (**), *p* < 0.001 (***), and *p* < 0.0001 (****). “ns” was used to indicate a lack of statistical significance.

## 3. Results

### 3.1. Effect of SERMs on SFTSV Infection In Vitro

Based on our previous study, the anti-SFTSV activities of 12 SERMs, 5 estrogen receptor degraders (ERDs), and 1 estrogen receptor agonist (ERA) were evaluated in Huh7 cells. In this study, T-705 was used as a positive anti-SFTSV drug.

**Figure 1:**
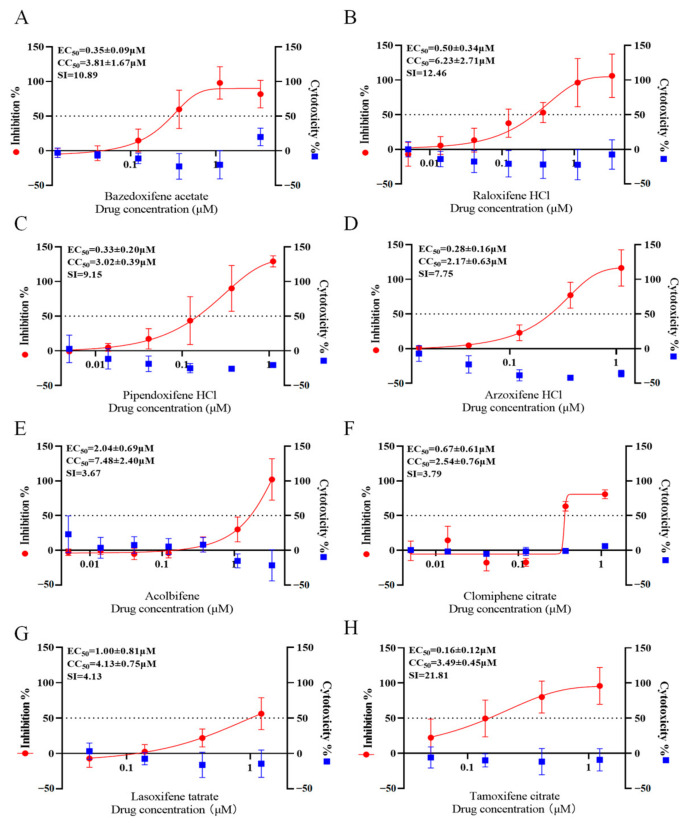
Selective estrogen receptor modulators (SERMs) inhibit severe fever with thrombocytopenia syndrome virus in the Huh7 cell line. The half-maximal effect concentration (EC_50_) and concentration of cytotoxicity 50% (CC_50_) of SERMs were calculated. Huh7 cell lines were co-cultured with gradient-diluted compounds and 100× TCID_50_ severe fever with thrombocytopenia syndrome virus for 5–7 days. The left or right y-axes represent the mean % CPE inhibition or cytotoxicity of the drug, respectively. Data were obtained from at least three independent tests. As shown in the figure, (**A**–**H**) represents eight SERMs, respectively.

The results show that eight SERMs possessed antiviral activities (Table 1 and Figure 1). In addition, the active compounds were evaluated in Huh7.5 cells with reduced interferon response due to the RIG-I mutation (Table S2). Interestingly, this inhibitory effect was abolished in Huh 7.5 cells, suggesting that the antiviral effect of SERMs may be related to the host’s innate immune response. ERAs and ERDs did not show any antiviral activity, suggesting that their antiviral activity may not be related to estrogen receptor activation or antagonism. In conclusion, SERMs, rather than ER degraders or agonists, exhibited potent anti-SFTSV activity.

### 3.2. SERMs Inhibit SFTSV RNA Replication and Infectious Virus Proliferation at Nontoxic Dose

**Figure 2:**
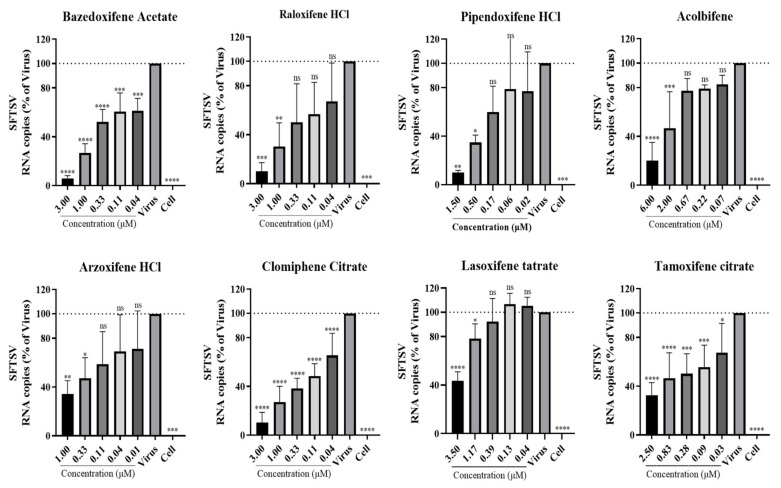
Selective estrogen receptor modulators significantly inhibit severe fever with thrombocytopenia syndrome virus RNA replication in vitro (quantitative real-time PCR). Huh7 cells were incubated with concentrations of test compounds or vehicle and then inoculated with severe fever with thrombocytopenia syndrome virus at an MOI of 0.1 for 48 h at 37 °C. The cells were treated with TRIzol reagent before quantitative real-time PCR assay. * *p* < 0.05, ** *p* < 0.01, *** *p* < 0.001, **** *p* < 0.0001; ns, no significance.

**Figure 3:**
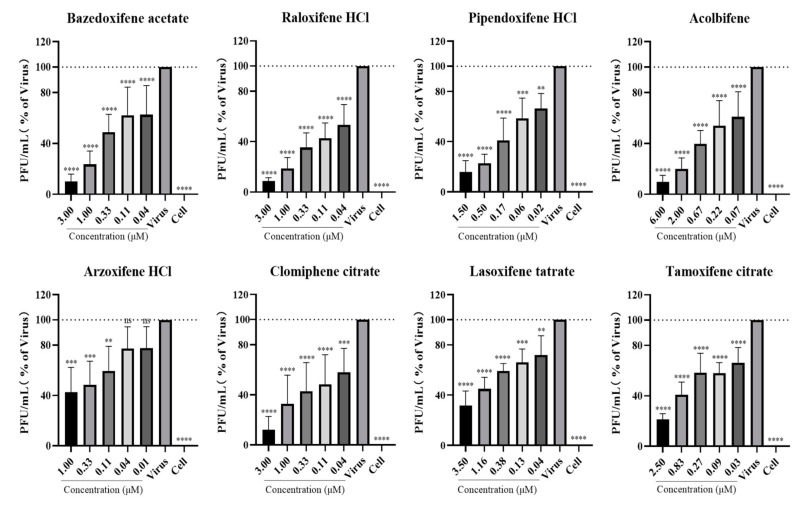
Inhibition effect of selective estrogen receptor modulators on the production of infectious severe fever with thrombocytopenia syndrome virus progeny virions in Huh7 cells. Cells were incubated with different compounds or vehicle (DMSO, dissolvent of the drugs) and inoculated with severe fever with thrombocytopenia syndrome virus at an MOI of 0.1 for 48 h at 37 °C. The supernatant was collected, and infectious viral particles were quantified with PFU assay. ** *p* < 0.01, *** *p* < 0.001, **** *p* < 0.0001.

We first evaluated the cytotoxicity of the SERMs using a CellTiter-Glo cell viability assay. As shown by the results (Figure S2), the compounds were not toxic to Huh7 cells at the tested concentrations. Subsequently, Huh7 cells were treated with different SERMs at nontoxic doses and inoculated with SFTSV for 48 h at 37 °C for RNA and PFU quantification. The results show that the SERMs exhibited a dose-dependent inhibition of both viral RNA replication (Figure 2) and infectious virus particle generation (Figure 3). Among them, the inhibitory effect of BZA and raloxifene (RLX) on SFTSV was relatively more significant, and approximately 90% of viral RNA and PFUs were inhibited at 3 μM. Although BZA and RLX are classified as benzothiophenes and phenylindoles, respectively, their molecular structures are similar (Figure S1).

In summary, these findings suggest that SERMs potently inhibit viral replication and the generation of infectious viral particles.

### 3.3. Anti-SFTSV Effect of BZA In Vivo

**Figure 4:**
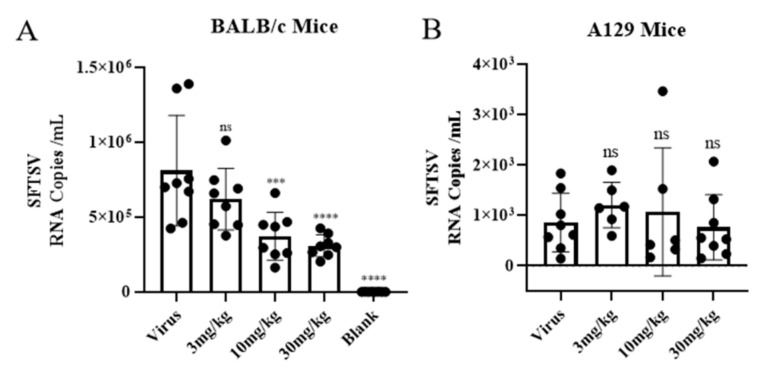
Bazedoxifene acetate (BZA) reduced severe fever with thrombocytopenia syndrome virus (SFTSV) viremia. Blood viral loads in SFTSV-infected Balb/c mice (**A**) and A129 mice (**B**) treated with BZA or vehicle were measured by quantitative real-time PCR. *** *p* < 0.001, **** *p* < 0.0001; ns, no significance.

BZA was selected for further in vivo evaluation because of its superior inhibitory effects in vitro. First, we evaluated its antiviral effect in the Balb/c adult mouse model to test the effects of BZA treatment (30 mg/kg, 10 mg/kg, and 3 mg/kg) on viremia (Figure 4A). The results show that BZA significantly reduced the viral load in Balb/c mice in a dose-dependent manner.

Considering that the antiviral effect of BZA in RIG-I mutated Huh7.5 cells was abolished, an A129 (Ifnar^−/−^) mouse model was exploited to verify the involvement of the innate immunity of BZA. The results show no significant difference in the viral load between the drug treatment groups and the viral group, further suggesting the possible involvement of the innate immune response in the antiviral mechanism of BZA (Figure 4B).

**Figure 5:**
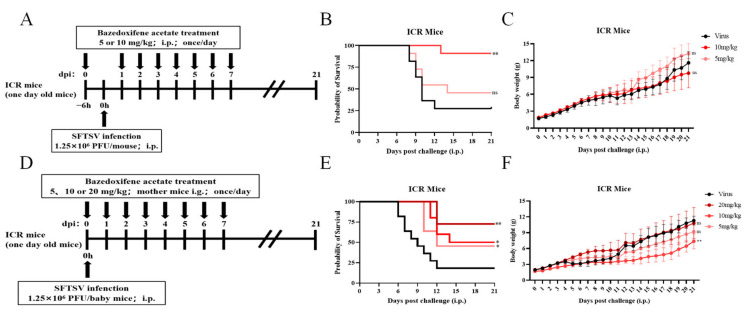
Bazedoxifene acetate (BZA) protected against severe fever with thrombocytopenia syndrome virus (SFTSV) challenge in vivo. (**A**,**D**) Schematic diagram of mice experiments. One-day-old ICR suckling mice were challenged with 1.25 × 10^6^ PFU SFTSV per mouse and administered with the indicated doses of BZA intraperitoneally (**B**,**C**) or by oral gavage to mother mice (**E**,**F**). The daily survival rate and changes in body weight of the mice were monitored for 21 days. Survival data were analyzed with a log-rank test. The data of weight curve were analyzed with unpaired, two-tailed *t*-tests. * *p* < 0.05, ** *p* < 0.01; ns, no significance.

In addition, an ICR lethal challenge model was used for the survival protection assay. For one-day-old suckling mice, 1.25 × 10^6^ PFU/suckling mice SFTSV was intraperitoneally inoculated, and different doses of BZA were administered once daily for seven consecutive days. Body weight and survival were measured for up to 21 dpi. Neonatal mice treated with 10 mg/kg of BZA showed 90.9% survival protection (Figure 5A–C). In addition, another set of experiments was conducted, but the mode of administration was changed to that of the mother mice (oral gavage). A total of 20 mg/kg of BZA was administered to adult mice rather than to suckling mice for comprehensive in vivo evaluation. Survival protection was 72.7% in neonatal mice whose mothers were treated with 20 mg/kg BZA (Figure 5D–F). BZA significantly protected the survival of SFTSV-infected suckling mice.

Collectively, these results suggest that BZA exerted potent therapeutical efficacy in vivo.

### 3.4. BZA Acted on the Post-Enter Stage of SFTSV Infection

**Figure 6:**
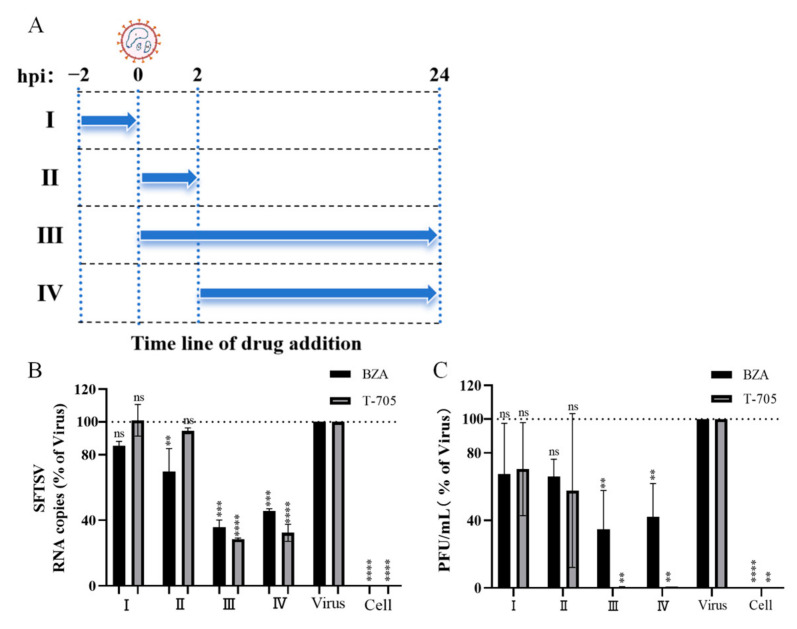
Bazedoxifene acetate interferes with severe fever with thrombocytopenia syndrome virus post-entry stage. (**A**) Scheme of time-of-drug-addition assay. Huh7 cells were seeded and cultured overnight. The virus (MOI = 0.1) was added at 0 h, and drugs were added at indicated time periods (I–IV) to inhibit different stages of the viral life cycle. The polymerase inhibitor T-705 was used as the positive control. (**B**,**C**) Intracellular viral RNA or infectious viral titers were quantified by quantitative real-time PCR or PFU, respectively, at 24 hpi. The percentage infection rate was calculated as the “drug-treated group/the virus group”. ** *p* < 0.01, *** *p* < 0.001, **** *p* < 0.0001; ns, no significance.

To evaluate the therapeutic effect of BZA during SFTSV infection, a time-of-drug-addition assay was performed, as previously described [22] (Figure 6A). After 24 h, the supernatant was collected for the PFU assay, and the cells were lysed with TRIzol reagent for qRT-PCR. The qRT-PCR and PFU results indicate that BZA had significant antiviral activity at the post-entry stage (Figure 6B,C). These results suggest that BZA may intervene at the intracellular stages of viral replication.

### 3.5. Transcriptome Analysis of BZA against SFTSV

**Figure 7:**
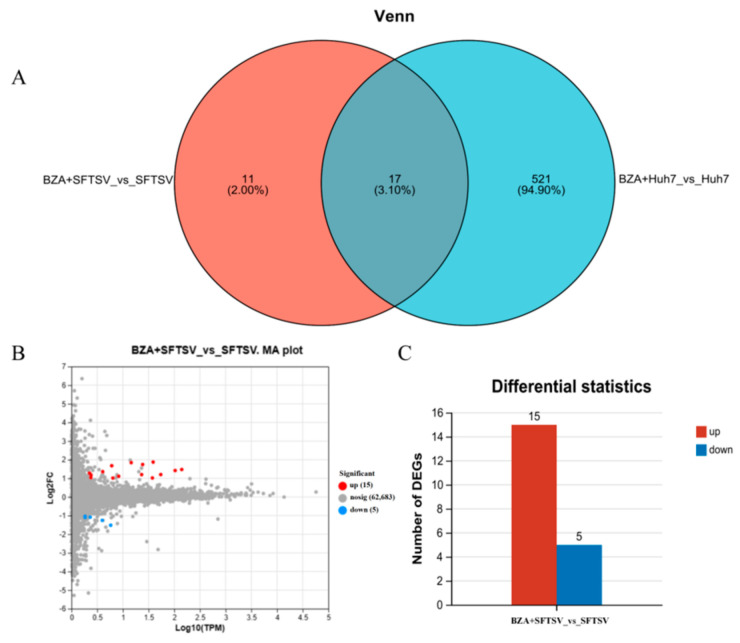
Transcriptome analysis of bazedoxifene acetate (BZA) against severe fever with thrombocytopenia syndrome virus (SFTSV). Huh7 cells were inoculated into 12-well plates at 1 × 10^5^ cells/well and cultured overnight. Subsequently, cells were infected with 0.1 MOI of SFTSV with the presence of 3 μM of BZA. At 24 hpi, cells were harvested to extract total RNA and subjected to transcriptome analysis. (**A**) The common and unique differentially expressed genes were identified by a Venn diagram. (**B**) Minus-versus-add plot identified the complex upregulation and downregulation. (**C**) The number of differentially expressed genes (DEGs).

**Figure 8:**
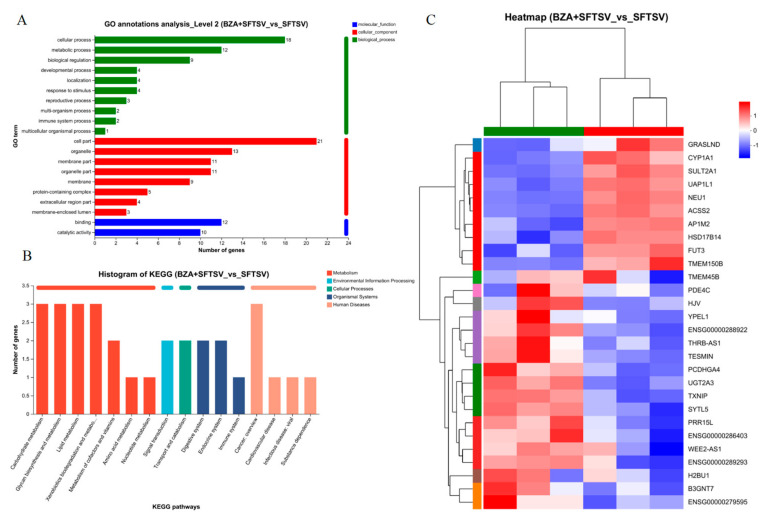
Top enriched GO annotation analysis (**A**) and KEGG enrichment analysis (**B**) for differentially expressed genes that were upregulated by severe fever with thrombocytopenia syndrome virus infection and restored by bazedoxifene acetate (BZA) treatment. (**C**) The heatmap of key DEGs related to severe fever with thrombocytopenia syndrome virus infection and BZA intervention were visualized. The color shows the fold change of detected genes.

To explore the potential molecular changes underlying the antiviral effect of BZA, we analyzed the transcriptome profiles of Huh7 cells at 24 h post-SFTSV infection in the presence and absence of BZA. Venn analysis was conducted to explore the correlation between BZA + SFTSV vs. SFTSV and BZA + Huh7 vs. Huh7 groups, and 17 differentially expressed genes (DEGs) were identified (Figure 7A). We used a minus-versus-add (MA) plot (Figure 7B) to identify the complex upregulation and downregulation that occurred following BZA treatment. Fifteen upregulated and five downregulated DEGs were observed in the BZA + SFTSV group compared with that in the SFTSV group (Figure 7C). According to the GO annotation analysis, the filtered DEGs in the BZA-treated group were defined as having various functions, including molecular function, cellular components, and biological processes (Figure 8A), and 16 significantly enriched KEGG pathways were identified (Figure 8B). After that, the heat map showed the specific DEGs (Figure 8C). Interestingly, GRASLND and CYP1A1 genes were upregulated, and TMEΜ45B and TXNIP genes were downregulated. In addition, we also analyzed the same genes implicated by the comparison of BZA + SFTSV vs. SFTSV to the comparison of Huh7 + BZA vs. Huh7. Clearly, some genes recur, such as TXNIP and CYP1A1, which seems to increase the possibility that they will be used as antiviral targets (Figure S3).

GRASLND affects interferon signaling potentially by binding to EIF2AK2. After silencing GRASLND, multiple IFN-mediated antiviral factors were upregulated (such as MX2, IFI44, IFI6, IFIT1, STAT1, et al.) [23]. CYP1A1 belongs to the cytochrome P450 (CYP) family. A study showed that by the downregulation of the AHR/CYP1A1 pathway, coronavirus infection could be reduced [24]. TMEM45B is a membrane protein that was identified as a novel interferon-stimulated antiviral factor against the Sindbis virus [25]. TXNIP is a redox protein that regulates pancreatic β-cell functions. Recent studies have shown that the overexpression of TXNIP reduces influenza virus infection, suggesting that TXNIP is an antiviral gene [26]. These results provide insights into the mechanism underlying the antiviral effects of BZA and provide new directions for the search for novel anti-SFTSV targets.

## 4. Discussion

*Bunyaviricetes* is the largest group of RNA viruses and comprises 2 orders and 15 families, including a large group of emerging and re-emerging viruses. These viruses can infect various species worldwide, including arthropods, protozoans, plants, animals, and humans, and pose a substantial threat to public health [27]. SFTSV, which is primarily transmitted by ticks, was first identified in China in 2009 [2,3,4,5,6]. A national assessment of the epidemiology of SFTS in China revealed high-risk areas in mid-eastern China [28]. Although ribavirin and favipiravir can inhibit SFTSV infection both in vitro and in vivo, their limited effect on patients with severe infections suggests an urgent need for novel antiviral agents [11,12,29].

In recent years, it has been found that SERMs may have potential antiviral effects against SARS-CoV-2, EBOV, and others. Existing studies show that none of these antiviral activities are achieved through the classical estrogen receptor signaling pathway. This suggests that the antiviral effects of SERMs may be off-target. However, the antiviral effect of SERMs on Bunyavirus remains to be explored [18,19,20].

In this study, we screened the in vitro anti-SFTSV activity of estrogen receptor-related compounds using a CPE assay. The results show that some SERMs exerted potent protective effects against virus-induced CPE. We selected eight SERMs for further antiviral evaluation. We first validated the cytotoxicity of SERMs in the selected concentration range to exclude cytotoxicity in subsequent experiments. All eight SERMs showed dose-dependent anti-SFTSV activity in terms of RNA and PFU inhibition. BZA is the latest generation of SERM, and we chose BZA for further antiviral assessment in mouse models. BZA reduced the viral load in the blood of adult Balb/c mice and significantly protected the survival of one-day-old suckling mice. However, there was no protection observed in type I interferon receptor-deficient A129 mice. This suggests that BZA has reduced antiviral activity in immunodeficient mice, implying that the anti-SFTSV activity of BZA may be related to innate immunity. In addition, we used time-of-addition assays to identify that BZA acted during the post-entry stage of the viral infection. However, BZA has been reported to exert antiviral activity against SARS-CoV-2 and Ebola virus during the entry stage of viral infection [18,19,20]. In time-of-addition assays, no significant inhibitory effect of BZA on SFTSV was observed at the entry stage, which we hypothesized might be due to the fact that the viral receptor of SFTSV is different from that of SARS-CoV-2 and Ebola virus.

Further, transcriptome analysis reveals that GRASLND, CYP1A1, TMEM45B, TXNIP, and other genes may participate in the antiviral process. These genes were previously reported to be associated with innate immunity, further confirming the correlation between the antiviral activity of BZA and the innate immune response. It provides insights into the mechanisms underlying the antiviral effects of BZA. However, our work thus far on SERMs against SFTSV should be considered exploratory. It is not possible to fully identify whether certain genes are involved in the antiviral response based only on a few changes in gene expression measured by RNA-seq. We have only proposed a few hypotheses that may serve as antiviral target genes. Therefore, in the future, it will be important to demonstrate their effects on viral replication through experiments, such as knockdown or overexpression.

In summary, SERMs represent a novel class of anti-SFTSV compounds. This study selected the third-generation SERM BZA by screening the in vitro anti-SFTSV activity of SERMs and further conducting a systematic pharmacodynamic evaluation and exploration of its action mechanisms. It was found that BZA can effectively inhibit SFTSV infection by regulating the host’s innate immune response both in vivo and in vitro. These results may provide novel core structures for future anti-SFTSV drug designs. At the same time, the study also provides a new option for discovering other important antiviral drugs for the Bunyavirus.

## Figures and Tables

**Table 1:** Anti-severe fever with thrombocytopenia syndrome virus activity and cytotoxicity of selective estrogen receptor modulators in Huh7 cells.

Functional Category	Compounds	EC_50_^a^ (μM) *	CC_50_^b^ (μM)	SI ^c^
Positive drug	T-705	11.41 ± 7.83	>100	>8.76
Selective estrogen receptor modulators (SERMs)	Bazedoxifene acetate	0.35 ± 0.09	3.81 ± 1.67	10.89
	Raloxifene HCl	0.50 ± 0.34	6.23 ± 2.71	12.46
	Pipendoxifene HCl	0.33 ± 0.20	3.02 ± 0.39	9.15
	Arzoxifene HCl	0.28 ± 0.16	2.17 ± 0.63	7.75
	Acolbifene HCl	2.04 ± 0.69	7.48 ± 2.40	3.67
	Clomiphene citrate	0.67 ± 0.61	2.54 ± 0.76	3.79
	Lasofoxifene tartrate	1.00 ± 0.81	4.13 ± 0.75	4.13
	Tamoxifen citrate	0.16 ± 0.12	3.49 ± 0.45	21.81
	Endoxifene HCl	>100	3.15 ± 0.55	-
	Ospemifene	>100	18.11 ± 5.48	-
	Cyclofenil	>100	19.37 ± 6.23	-
	Chlorotrianisene	>100	>100	-
Estrogen receptor degraders (ERDs)	Fulvestrant	>100	>100	-
	Amcenestrant	>100	7.67 ± 1.39	-
	Brilanestrant	>100	11.87 ± 0.95	-
	Camizestrant	>100	49.48 ± 9.61	-
	Estriol	>100	67.27 ± 20.11	-
Estrogen receptor agonists (ERAs)	G-1	>100	0.46 ± 0.21	-

* A viral infection dose of 100 × TCID_50_ was used in this experiment. The TCID_50_ used in this study was determined on Huh7 cells. ^a^ EC_50_ = Half-maximal effective concentration. ^b^ CC_50_ = Half-maximal cytotoxic concentration. ^c^ SI = Selectivity index; CC_50_/EC_50._

## Data Availability

Data are contained within the article.

## Supplementary Materials

The following supporting information can be downloaded at: https://www.mdpi.com/article/10.3390/v16081332/s1, Figure S1: The chemical structure of selective estrogen receptor modulators; Figure S2: Cytotoxicity of selective estrogen receptor modulators in Huh7 cell line. Huh7 cells were treated with selective estrogen receptor modulators for 48 h before the cytotoxicity was evaluated using CellTiter-Glo cell viability assay; Figure S3: The heatmap of key DEGs showed the same genes implicated by the comparison of Bazedoxifene acetate (BZA) + severe fever with thrombocytopenia syndrome virus (SFTSV) vs. SFTSV to the comparison of Huh7 + BZA vs. Huh7. The color shows the fold change of detected genes; Table S1: Compounds for screening of antiviral effects against SFTSV in vitro; Table S2: Anti-severe fever with thrombocytopenia syndrome virus activity and cytotoxicity of selective estrogen receptor modulators in Huh7.5 cells.
