# Supplementary material for: Antiviral Activity of Selective Estrogen Receptor Modulators against Severe Fever with Thrombocytopenia Syndrome Virus In Vitro and In Vivo
Source: Viruses. 2024 Aug 20;16(8):1332. doi: 10.3390/v16081332 (PMC11360069; doi:10.3390/v16081332)
Supplement: Supplementary file 1 [file viruses-16-01332-s001.zip › viruses-3103568-supplementary.pdf]

viruses MDPI

Supplementary Materials:

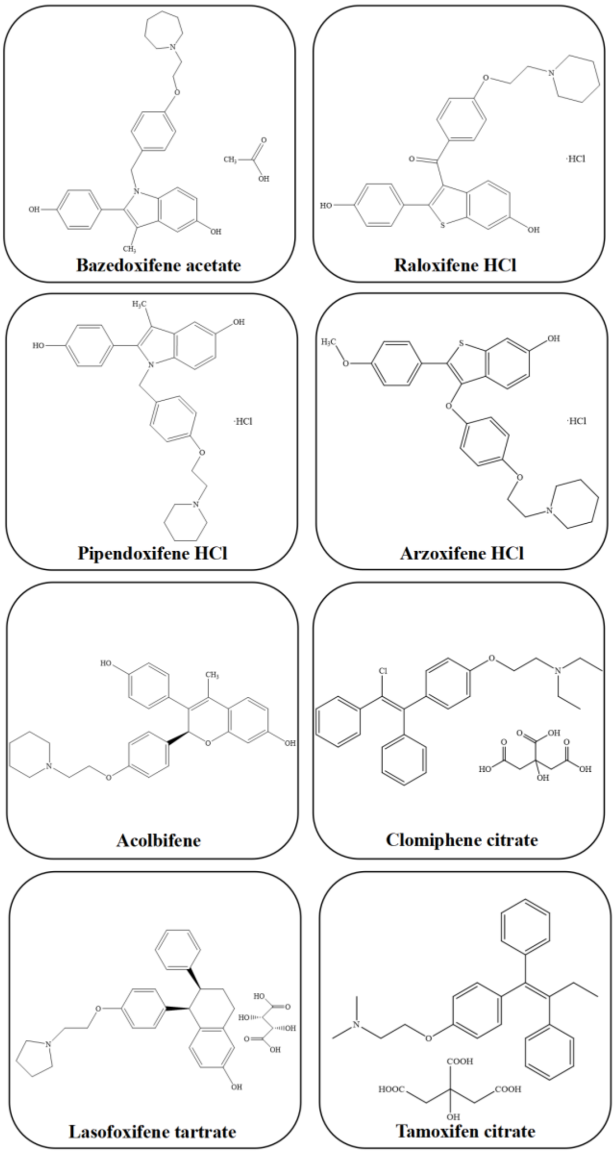

Figure S1. The chemical structure of selective estrogen receptor modulators.

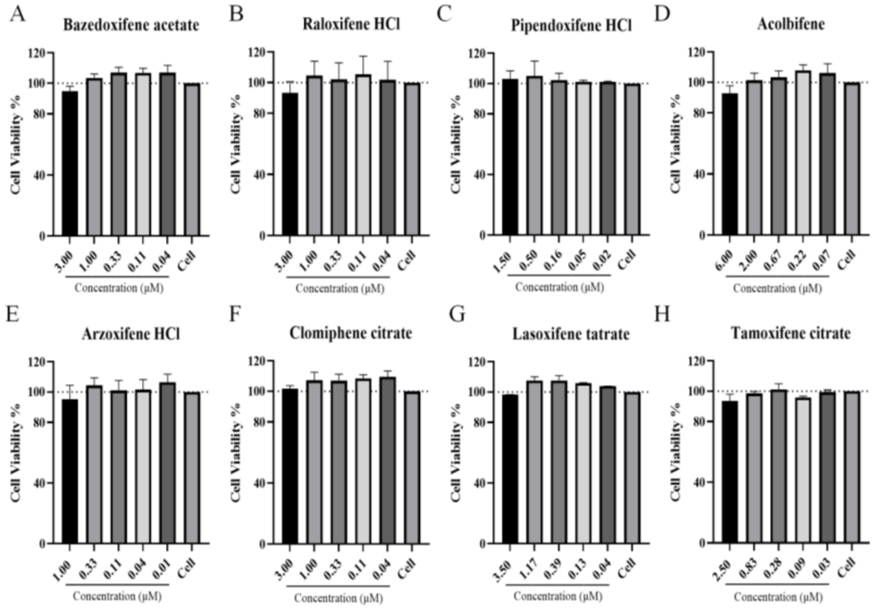

Figure S2. Cytotoxicity of selective estrogen receptor modulators in Huh7 cell line. Huh7 cells were treated with selective estrogen receptor modulators for 48 h before the cytotoxicity was evaluated using CellTiter-Glo cell viability assay.

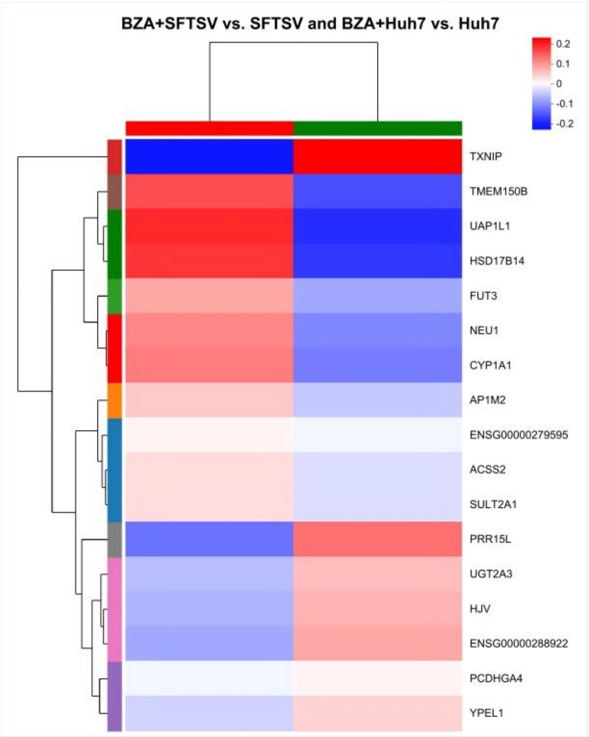

Figure S3. The heatmap of key DEGs showed the same genes implicated by the comparison of Bazedoxifene acetate (BZA) + severe fever with thrombocytopenia syndrome virus (SFTSV) vs. SFTSV to the comparison of Huh7 + BZA vs. Huh7. The color shows the fold change of detected genes.

Table S1. Compounds for screening of antiviral effects against SFTSV *in vitro*.

Supplier	Compound name	Catalog no.
MedChemExpress (Shanghai, China)	Acolbifene HCl	HY-16023
	Arzoxifene HCl	HY-13556A
	Favipiravir (T-705)	HY-14768
TargetMol Chemicals (Shanghai, China)	Lasoxifene tatrate	T7839
	Amcenestrant	T12832
	Brilanestrant	T5118
	Clomiphene citrate	T1193
	Fulvestrant	T2146
	Cyclofenil	T8349
	Chlorotrianisene	T2569
	Tamoxifen citrate	T6906
	Estriol	T1571
	Pipendoxifene HCl	T12482
Selleck Chemicals (Shanghai, China)	Raloxifene HCl	S1227
	G-1	S0851
	Camizestrant	S8958
	Endoxifene HCl	S7839
	Bazedoxifene acetate	S2167

Table S2. Anti-severe fever with thrombocytopenia syndrome virus activity and cytotoxicity of selective estrogen receptor modulators in Huh7.5 cells.

Compounds	EC_{50}[a] (μM)*	CC_{50}[b] (μM)	SI[c]
T-705	43.49±14.24	＞200	＞4.48
Bazedoxifene acetate	＞100	2.79±0.52	-
Raloxifene HCl	＞100	6.01±0.10	-
Arzoxifene HCl	＞100	2.01±0.08	-
Acolbifene HCl	＞100	3.78±0.01	-
Lasoxifene tatrate	＞100	3.66±0.05	-
Tamoxifene citrate	＞100	3.73±0.04	-
Clomiphene	＞100	16.76±0.02	-

*A viral infection dose of 100 × $TCID_{50}$ was used in this experiment. The $TCID_{50}$ used in this study was determined on Huh7.5 cells.

[a]EC_{50}=Half-maximal effective concentration

[b]CC_{50}=Half-maximal cytotoxic concentration

[c]SI=Selectivity index; CC_{50}/EC_{50}

Disclaimer/Publisher's Note: The statements, opinions and data contained in all publications are solely those of the individual author(s) and contributor(s) and not of MDPI and/or the editor(s). MDPI and/or the editor(s) disclaim responsibility for any injury to people or property resulting from any ideas, methods, instructions or products referred to in the content.
